# Association of temporal change in body mass index with sudden cardiac arrest in diabetes mellitus

**DOI:** 10.1186/s12933-024-02130-4

**Published:** 2024-01-28

**Authors:** Joo Hee Jeong, Yun Gi Kim, Kyung-Do Han, Seung-Young Roh, Hyoung Seok Lee, Yun Young Choi, Jaemin Shim, Jong-Il Choi, Young-Hoon Kim

**Affiliations:** 1 Division of Cardiology, Department of Internal Medicine, Korea University College of Medicine, Korea University Anam Hospital, 73 Goryeodae-ro, Seongbuk-gu, 02841 Seoul, Republic of Korea; 2 Department of Statistics and Actuarial Science, Soongsil University, Seoul, Republic of Korea; 3 Division of Cardiology, Department of Internal Medicine, Korea University College of Medicine, Korea University Guro Hospital, Seoul, Republic of Korea

**Keywords:** Underweight, Diabetes mellitus, Sudden cardiac arrest

## Abstract

**Background:**

Underweight imposes significant burden on cardiovascular outcomes in patients with diabetes mellitus. However, less is known about the impact of serial change in body weight status measured as body mass index (BMI) on the risk of sudden cardiac arrest (SCA). This study investigated the association between SCA and temporal change in BMI among patients with diabetes mellitus.

**Methods:**

Based on Korean National Health Insurance Service database, participants with diabetes mellitus who underwent health examination between 2009 and 2012 and had prior health examination data (four years ago, 2005–2008) were retrospectively analyzed. BMI was measured at baseline (2005–2008) and 4-year follow-up health examination (2009–2012). Patients were classified in four groups according to the body weight status and its temporal change: sustained non-underweight, sustained underweight, previous underweight, and newly developed underweight. Primary outcome was defined as occurrence of SCA.

**Results:**

A total of 1,355,746 patients with diabetes mellitus were included for analysis, and SCA occurred in 12,554 cases. SCA was most common in newly developed underweight (incidence rate = 4.45 per 1,000 person-years), followed by sustained underweight (incidence rate = 3.90), previous underweight (incidence rate = 3.03), and sustained non-underweight (incidence rate = 1.34). Adjustment of covariates resulted highest risk of SCA in sustained underweight (adjusted hazard ratio = 2.60, 95% confidence interval [2.25–3.00], sustained non-underweight as a reference), followed by newly developed underweight (2.42, [2.15–2.74]), and previous underweight (2.12, [1.77–2.53]).

**Conclusions:**

In diabetes mellitus, sustained underweight as well as decrease in body weight during 4-year follow-up imposes substantial risk on SCA. Recovery from underweight over time had relatively lower, but yet increased risk of SCA. Both underweight and dynamic decrease in BMI can be associated with increased risk of SCA.

**Supplementary Information:**

The online version contains supplementary material available at 10.1186/s12933-024-02130-4.

## Background

Body mass index (BMI) is the most common metric to assess individual’s degree of obesity. It also reflects nutritional status and muscle mass that correlates with functional capacity. Previous landmark studies on BMI have identified a J-shaped association between BMI and mortality: underweight and obesity are both associated with increased risk of mortality [1, 2]. Underweight, defined as BMI less than 18.5 kg/m^2^, is known to have increased risk of mortality and various cardiovascular adverse events [3–5]. In patients with acute myocardial infarction, underweight was identified as an independent risk factor for mortality [6]. Similarly, analysis of national registry of implantable cardioverter-defibrillator revealed increased risk of mortality and morbidities in underweight recipients [7]. Diabetes mellitus is a strong risk factor for atherosclerotic cardiovascular disease and sudden cardiac arrest (SCA) [8–10]. Presence of diabetes mellitus as well as hyperglycemia has 1.7 to 3.2-fold increased risk of SCA [11, 12]. In patients with diabetes mellitus, underweight was associated with 2.4-fold increased risk of SCA [5]. Also, patients with diabetes mellitus can be more vulnerable to underweight or decrease in body weight.

Body weight status can be highly variable across time, and cross-sectional data of BMI measured at a certain period may be limited to establish its association with SCA. There may be significant differences between patients who have maintained underweight consistently, and those who experienced substantial loss of body weight. Fluctuation of body weight status was reported to increase mortality in patients with coronary artery disease [13]. In patient with diabetes mellitus and high risk of cardiovascular diseases, extremes of weight gain as well as weight loss are associated with increased risk of heart failure and cardiovascular death [14]. However, less is known about the temporal change of body weight status and the risk of SCA in diabetes mellitus. Therefore, based on the nationwide health examination cohort of patients with diabetes mellitus, we aimed to investigate the association between SCA and temporal change in BMI and examine the influence of body weight change on SCA.

## Methods

### Database

This study is based on the Korean National Health Insurance Service (K-NHIS) database, which include exclusively all citizens of Republic of Korea. The K-NHIS provides medical insurance benefit to whole population, regardless of individual’s solvency [15]. In order to detect disease and treat in earlier phase, the K-NHIS provides regular health examinations and cancer screening program [15]. Among the K-NHIS database, nationwide health examination data was obtained for analysis. Nationwide health examination is provided once in every two years – that includes (i) self-reported questionnaires of lifestyles and behaviors, (ii) physical examination such as body weight, height, waist circumference, blood pressure measurement, and (iii) laboratory testing of blood and urine [16]. In the health examination data, medical records based on diagnostic codes of International Classification of Disease, 10th revision (ICD-10) and drug prescription records are also included. This study was approved by Institutional Review Board of Korea University Medicine Anam Hospital and official review committee of the K-NHIS. This study conformed to the principle of 2013 Declaration of Helsinki. Written informed consent was waived by the Institutional Review Board of Korea University Medicine Anam Hospital due to the retrospective nature of this study.

### Study population

**Fig. 1:**
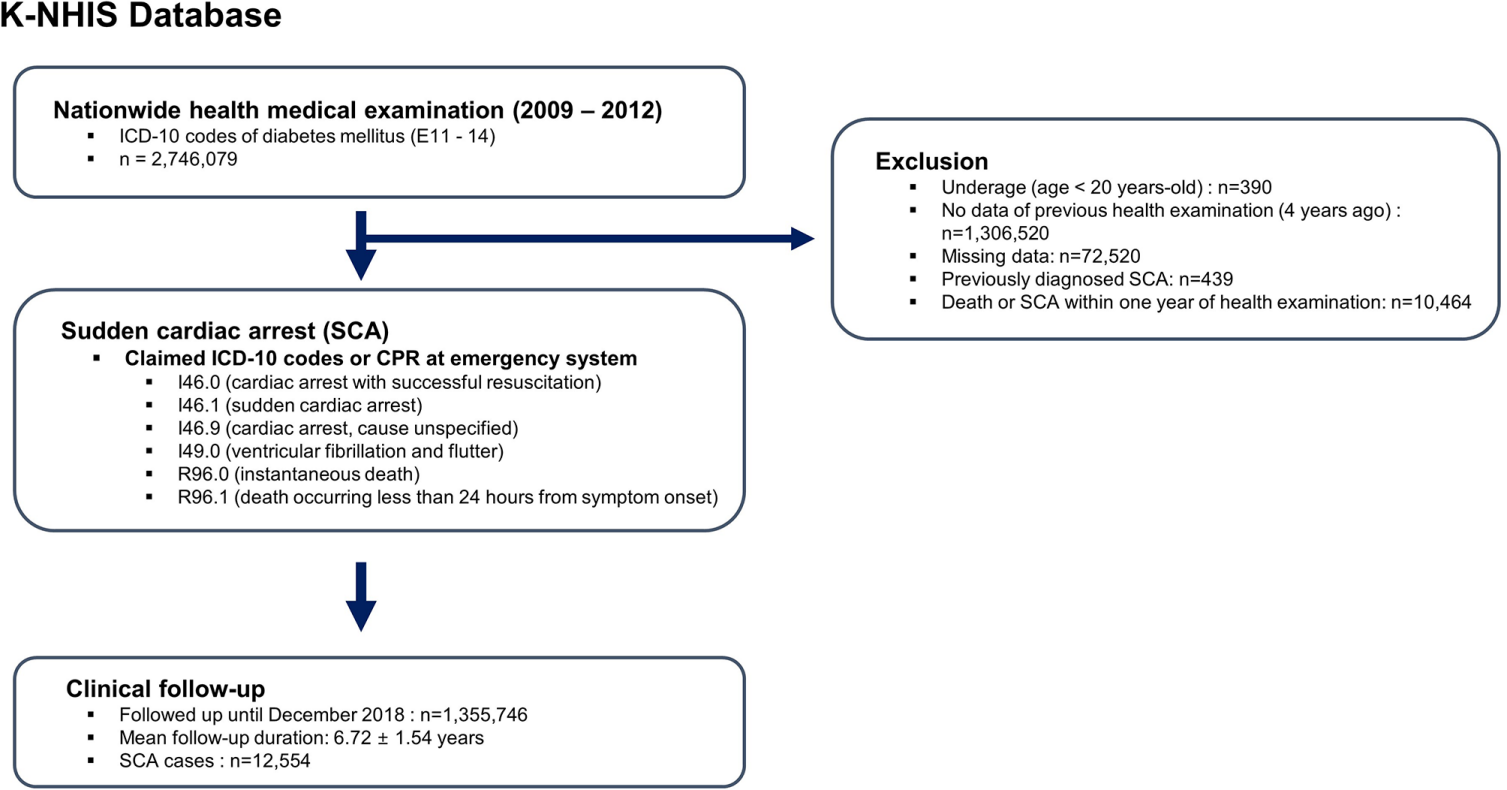
Flowsheet of the study K-NHIS: Korean National Health Insurance Service; ICD-10: International Classification of Disease, 10th revision; SCA: sudden cardiac arrest; CPR: cardiopulmonary resuscitation

Data from participants who underwent health examination from 2009 to 2012 was extracted, and those with history of diabetes mellitus by ICD-10 code (E11-14) were screened (*n* = 2,746,079, Fig. 1). Only participants who had undergone prior health examination four years ago (2005–2008) were included to enable analysis regarding temporal change in BMI. Therefore, health examinations performed during 2009 to 2012 were follow-ups and examinations performed in 2005 to 2008 were baseline examinations. Following patients were excluded for analysis: (i) participants who were under 20 years (*n* = 390); (ii) participants who did not undergo baseline health examinations at 2005–2008 (*n* = 1,306,520); (iii) participants with missing data (*n* = 72,520); (iv) participants with previous diagnosis of SCA (*n* = 439); and (v) participants who experienced SCA or died within one year after health examination at 2009–2012 (*n* = 10,464).

Clinical observation period was from health examination at 2009–2012 to December 2018. There were no follow-up losses except for death and emigrations. Baseline medical history such as hypertension, diabetes mellitus, or prior SCA events was identified by the data obtained during January 2002 to December 2008.

Patients were divided into four groups regarding their baseline body weight status and its change at 4-year follow-up. Patients who were not in underweight at baseline and nor after four years were defined as ‘sustained non-underweight’. Patients who were not in underweight at baseline but were in underweight four years later were defined as ‘newly developed underweight’. Patients who were in underweight at baseline as well as at four years later were defined as ‘sustained underweight’. Patients who were in underweight at baseline but no longer in underweight at four years later were defined as ‘previous underweight’. Body weight status measured at each health examination was further subdivided into three groups (underweight [BMI < 18.5], normal weight [18.5 ≤ BMI < 25], and overweight [BMI ≥ 25]) to compare the risk of SCA. Other definitions of variables are listed in Additional Table 1.

**Table 1:** Demographics according to temporal change of BMI

	Underweight (Pre / Post)				***p***-value
	No / No (*n* = 1,330,899) Sustained non-underweight	No / Yes (*n* = 10,270) Newly developed underweight	Yes / No (*n* = 6,415) Previous underweight	Yes / Yes (*n* = 8,162) Sustained underweight	
**Age, years**	58.5 ± 11.7	63.0 ± 13.5	57.4 ± 15.4	58.5 ± 15.4	< 0.001
**Male sex**	825,865 (62.1%)	5,759 (56.1%)	3,827 (59.7%)	5,225 (64.0%)	< 0.001
**Body mass index, kg/m** ^**2**^	25.1 ± 3.2	17.7 ± 0.8	20.1 ± 1.9	17.2 ± 1.0	< 0.001
**Income, lowest quartile**	234,673 (17.6%)	2,046 (19.9%)	1,296 (20.2%)	1,587 (19.4%)	< 0.001
**Smoking**					< 0.001
Non-smoker	742,871 (55.8%)	5,932 (57.8%)	3,513 (54.8%)	4,084 (50.0%)	
Ex-smoker	274,040 (20.6%)	1,407 (13.7%)	1,053 (16.4%)	1,096 (13.4%)	
Current smoker	313,988 (23.6%)	2,931 (28.5%)	1,849 (28.8%)	2,982 (36.5%)	
**Drinking**					< 0.001
Non-drinker	757,270 (56.9%)	6,873 (66.9%)	3,861 (60.2%)	4,845 (59.4%)	
Mild-drinker	451,410 (33.9%)	2,583 (25.2%)	2,055 (32.0%)	2,615 (32.0%)	
Heavy-drinker	122,219 (9.2%)	814 (7.9%)	499 (7.8%)	702 (8.6%)	
**Regular exercise**	299,873 (22.5%)	1,731 (16.9%)	1,073 (16.7%)	1,254 (15.4%)	< 0.001
**Hypertension**	772,899 (58.1%)	4,456 (43.4%)	2,545 (39.7%)	2,676 (32.8%)	< 0.001
**Dyslipidemia**	573,712 (43.1%)	2,783 (27.1%)	1,633 (25.5%)	1,486 (18.2%)	< 0.001
**Chronic kidney disease**	152,772 (11.5%)	1,363 (13.3%)	735 (11.5%)	765 (9.4%)	< 0.001
**Cardiovascular disease**	81,117 (6.1%)	896 (8.7%)	373 (5.8%)	402 (4.9%)	< 0.001
**Duration of diabetes mellitus ≥ 5 years**	453,736 (34.1%)	4,043 (39.4%)	1,962 (30.6%)	2,400 (29.4%)	< 0.001
**Use of insulin**	111,890 (8.4%)	1,638 (16.0%)	914 (14.3%)	980 (12.0%)	< 0.001
**Use of oral hypoglycemic agents, ≥3**	200,474 (15.1%)	1,925 (18.7%)	837 (13.1%)	1,019 (12.5%)	< 0.001
**Waist Circumference, cm**	85.7 ± 8.2	71.2 ± 6.6	74.8 ± 6.9	68.8 ± 5.7	< 0.001
**Systolic Blood Pressure, mmHg**	128.8 ± 15.3	123.0 ± 17.2	124.5 ± 16.5	121.7 ± 16.8	< 0.001
**Diastolic Blood Pressure, mmHg**	78.8 ± 10.0	75.2 ± 10.6	75.9 ± 10.3	74.9 ± 10.4	< 0.001
**Fasting glucose, mg/dL**	141.5 ± 43.5	148.2 ± 63.7	140.2 ± 43.8	144.0 ± 51.0	< 0.001
**Total cholesterol, mg/dL**	194.8 ± 41.9	183.9 ± 42.6	188.2 ± 40.0	182.0 ± 37.5	< 0.001
**HDL -cholesterol, mg/dL**	51.6 ± 22.5	58.4 ± 26.3	57.2 ± 25.0	61.0 ± 28.0	< 0.001
**LDL -cholesterol, mg/dL**	110.3 ± 40.3	102.6 ± 41.5	106.1 ± 37.7	100.0 ± 37.9	< 0.001
**Triglyceride, mg/dL**	144.8 (144.7–145.0)	102.3 (101.2–103.4)	110.4 (108.9–111.9)	95.6 (94.5–96.7)	< 0.001

Data were from follow-up health examinations performed during 2009 to 2012

BMI: body mass index; HDL: high-density lipoprotein; LDL: low-density lipoprotein

### Outcome measurement

Primary outcome was defined as occurrence of SCA event during follow-up, which included ICD-10 codes of following: ‘cardiac arrest with successful resuscitation (I46.0)’, ‘sudden cardiac arrest (I46.1)’, ‘cardiac arrest, cause unspecified (I46.9)’, ‘ventricular fibrillation and flutter (I49.0)’, ‘instantaneous death (R96.0)’, and ‘death occurring less than 24 hours from symptom onset (R96.1)’. Out-of-hospital cardiac arrest declared at emergency department was defined as SCA event, and events during in-hospital admission were not included. Performance of cardiopulmonary resuscitation at emergency department without ICD-10 codes for SCA was also classified as SCA event and aborted sudden cardiac death events were also defined as SCA. In order to differentiate non-cardiac causes of sudden arrest with SCA, patients with following diagnosis within 6 months of SCA event were excluded – hemorrhagic stroke, ischemic stroke, asphyxia, suffocation, drowning, anaphylaxis, gastrointestinal bleeding, major trauma, sepsis, hit by lightning, electric shock, or burn. Also, people with prior SCA events that occurred before start of clinical follow-up (health examination at 2009–2012) were excluded. In addition, since the claim of ICD-10 codes for SCA that occurred immediately after follow-up health examination (at 2009–2012) can be actual SCA event after health examination or just repeat claim of prior SCA that occurred before health examination, the claims for SCA that occurred within one year after health examination (at 2009–2012) was not counted as a main outcome. Therefore, occurrence of SCA was tracked from 2010 to 2013 (one year after health examination performed in 2009–2012) to December 2018. Outcome measurement and variable definitions in this study was validated in previous studies [9, 17–20]. 

### Statistical analysis

Categorical variables are expressed as number and percentage, and continuous variables were expressed as means and standard deviations, or medians and quartiles as appropriate. Student’s t-test and Chi-square test was used for comparison of continuous and categorical variables, respectively. The incidence of SCA was calculated as event numbers per 1,000 person-years of follow-up. Kaplan-Meier analysis and log-rank t-test were used to assess the influence of time-dependent variables. Cox-proportional hazards model was used to calculate hazards ratios (HR) and 95% confidence intervals (CI). Multivariate cox-regression analysis was done to adjust covariates: (i) model 1 (unadjusted model), (ii) model 2 adjusted with age and sex, (iii) model 3 adjusted with age, sex, income (quartile), smoking, drinking, regular exercise, hypertension, dyslipidemia, chronic kidney disease, and cardiovascular disease, and (iv) model 4 adjusted with age, sex, income (quartile), smoking, drinking, regular exercise, hypertension, dyslipidemia, chronic kidney disease, cardiovascular disease, fasting glucose level, duration of diabetes mellitus, use of insulin, and use of multiple oral hypoglycemic agents. Demographics, physical factors, social habits, or comorbidities that could confound the association of body weight status and outcome were included in multivariate analysis, which were obtained from baseline health examination (2005–2008) [21, 22]. We identified covariates that showed significant difference between people who experienced and did not experience SCA during follow-up and included such variables into our multivariate model. In order to assess the influence of body weight change, adjusted risk of SCA were compared between different body weight status at 4-year follow-up health examination. Subgroup analysis was performed to compare the correlation of body weight change and risk of SCA among different subgroups. All tests were two-tailed, and statistical significance was defined as *p*-values ≤ 0.05. Statistical analyses were performed with SAS version 9.2 (SAS Institute, Cary, NC, USA).

## Results

### Study population

A total of 1,355,746 patients diagnosed as diabetes mellitus were included in analysis, and the mean follow-up period was 6.7 ± 1.5 years. Patients who were excluded due to lack of baseline health examination had younger age, and lower incidence of SCA (Additional Table 2). In the study population, most of the patients were classified as sustained non-underweight (98.2%, Table 1). Sustained non-underweight group had lower proportion of current smokers, and regular exercise was more prevalent. On the other hand, sustained non-underweight group featured higher proportion of mild- and heavy-drinkers, hypertension and dyslipidemia. Systolic blood pressure, diastolic blood pressure, total cholesterol, and low-density lipoprotein cholesterol were also higher in sustained non-underweight group. Likewise, newly developed underweight group reflected distinct characteristics of older age and more frequent chronic kidney disease and cardiovascular disease. Newly developed underweight group revealed more severe form of diabetes mellitus: longer duration (five years or longer), multiple oral hypoglycemic agents (≥ 3), insulin use, and higher fasting glucose.

**Table 2:** Impact of temporal change of BMI on SCA

Underweight	N	Event	Duration	Incidence rate	Adjusted HR (95% Confidence Interval)			
					Model 1	Model 2	Model 3	Model 4
**Sustained non-underweight**	1,330,899	11,968	8,964,343	1.335	1 (Reference)	1 (Reference)	1 (Reference)	1 (Reference)
**Newly developed underweight**	10,270	270	60,622	4.454	3.404 (3.017–3.840)	2.591 (2.296–2.924)	2.600 (2.303–2.936)	2.423 (2.146–2.736)
**Previous underweight**	6,415	123	40,639	3.027	2.287 (1.915–2.732)	2.176 (1.822–2.599)	2.182 (1.826–2.607)	2.120 (1.774–2.532)
**Sustained underweight**	8,162	193	49,486	3.900	2.968 (2.574–3.421)	2.581 (2.239–2.976)	2.638 (2.287–3.044)	2.601 (2.254–3.002)

Incidence rate is per 1,000 person-years

BMI: body mass index; SCA: sudden cardiac arrest; HR: hazard ratio

Model 1: non-adjusted

Model 2: adjusted for age and sex

Model 3: adjusted for age, sex, income, smoking status, alcohol consumption status, regular exercise, hypertension, dyslipidemia, chronic kidney disease, and cardiovascular disease

Model 4: adjusted for age, sex, income, smoking status, alcohol consumption status, regular exercise, hypertension, dyslipidemia, chronic kidney disease, cardiovascular disease, fasting glucose, duration of diabetes mellitus, use of insulin, and use of multiple (≥ 3) oral hypoglycemic agent

During follow-up, 12,554 cases of SCA occurred (Additional Table 3). Patients who experienced SCA were older (66.1 ± 10.5 vs. 58.5 ± 11.8 years) and revealed higher proportion of male sex (73.7 vs. 61.9%), lowest income quartile (20.1 vs. 17.7%), and current smokers (27.2 vs. 23.7%). In addition, hypertension (71.9 vs. 57.6%), chronic kidney disease (24.3 vs. 11.4%), cardiovascular disease (11.2 vs. 6.1%), diabetes mellitus with longer duration (five years or longer, 50.2 vs. 33.9%), and diabetes mellitus with insulin use or multiple oral hypoglycemic agent use (three or more, 21.6 vs. 15.0%) were more frequent in patients who experienced SCA during follow-up.

### Incidence and risk of sudden cardiac arrest

**Fig. 2:**
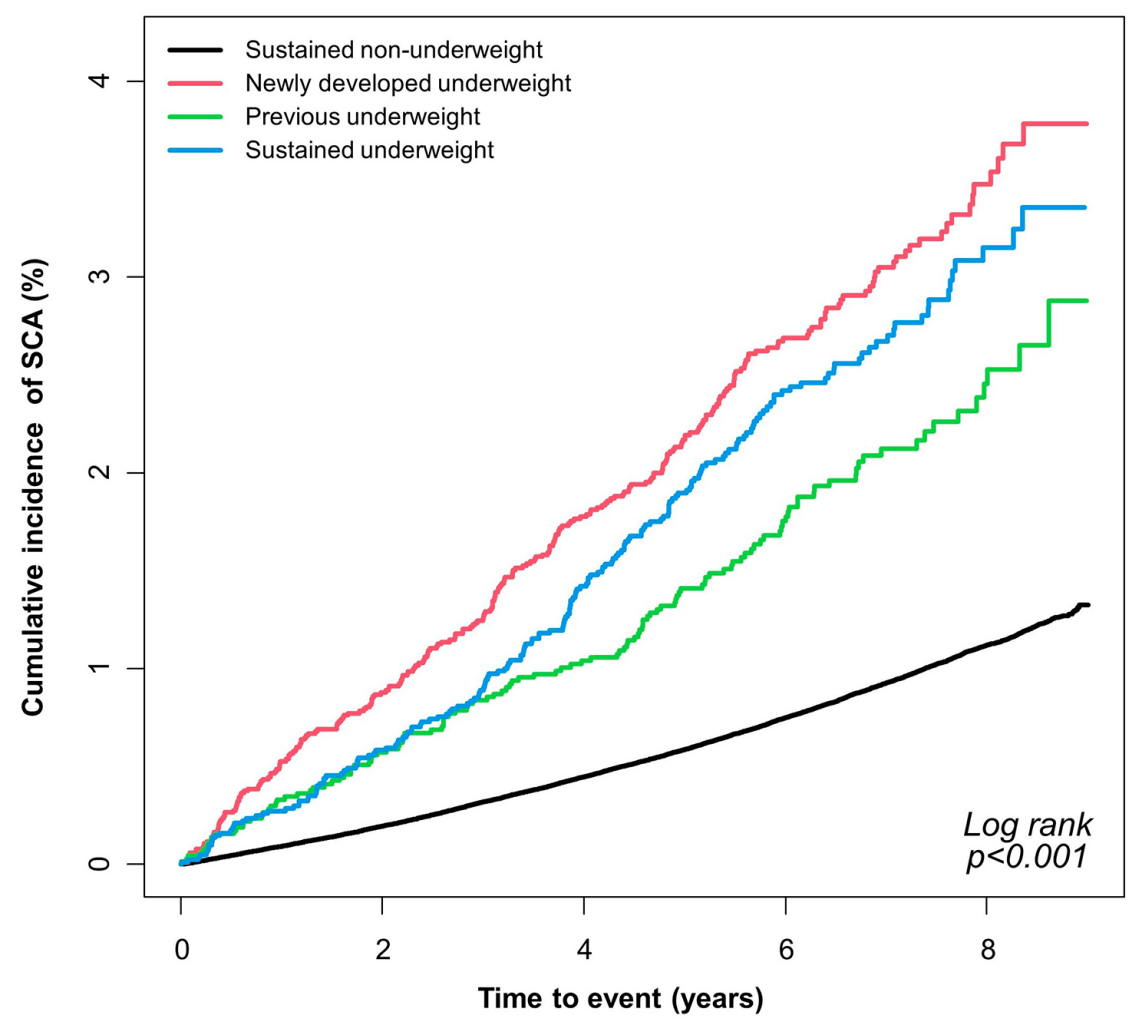
Cumulative incidence of SCA SCA: sudden cardiac arrest

Sudden cardiac arrest was most common in newly developed underweight (incidence rate = 4.454 per 1,000 person-years), followed by sustained underweight (incidence rate = 3.900) and previous underweight (incidence rate = 3.027, Table 2; Fig. 2). Further adjustment of social habit, comorbidities, and clinical factors related to severity of diabetes mellitus revealed highest risk of SCA in sustained underweight (adjusted-HR = 2.601; 95% CI = 2.254–3.002), followed by newly developed underweight (adjusted-HR = 2.423; 95% CI = 2.146–2.736), and previous underweight (adjusted-HR = 2.120; 95% CI = 1.774–2.532).

**Fig. 3:**
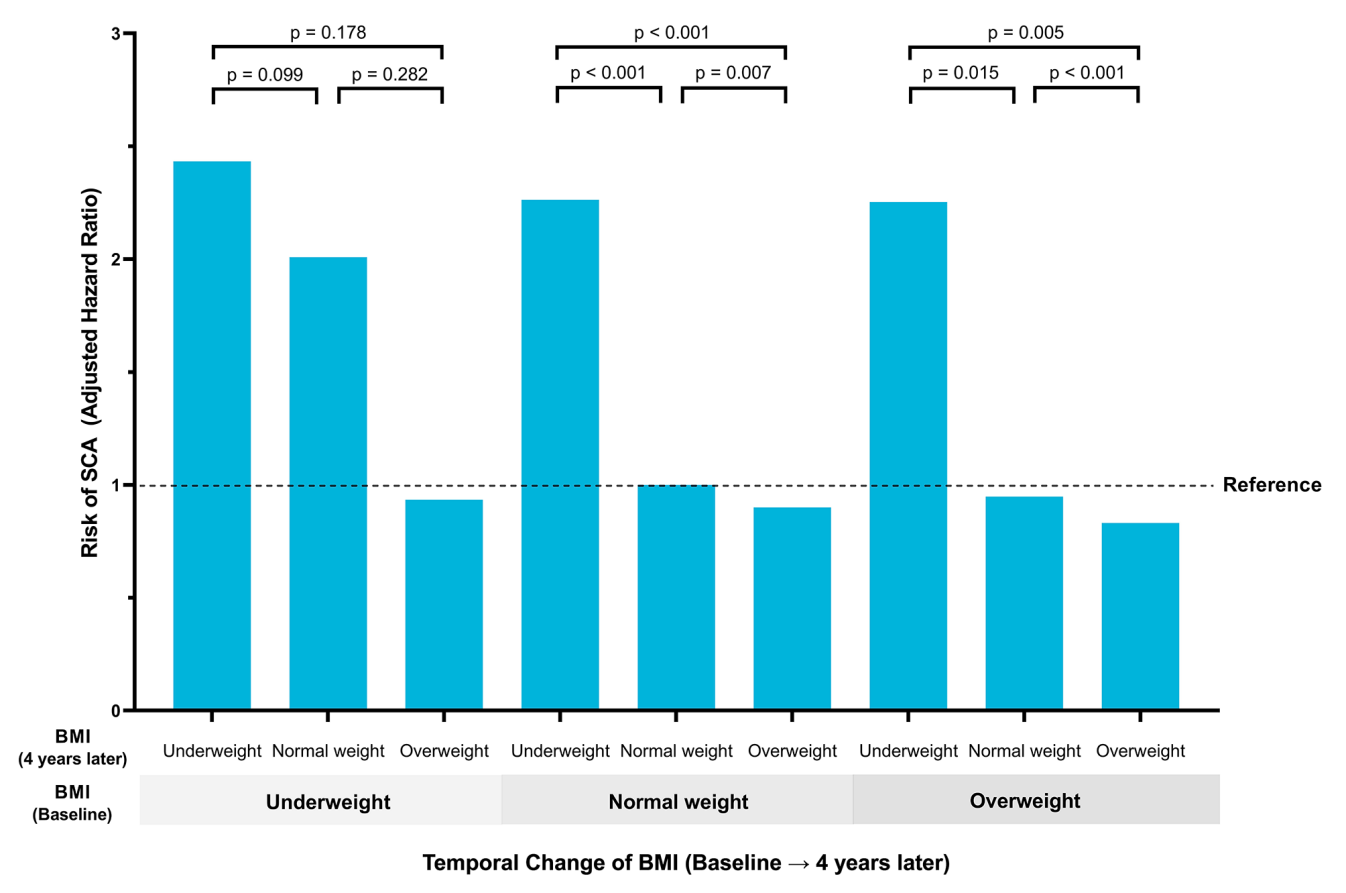
SCA risk according to change in BMI Risk of SCA was stratified by further subdivision of body weight change from baseline examination to 4 years follow-up. Underweight is defined as BMI < 18.5 (kg/m^2^), normal weight is defined as 18.5 ≤ BMI < 25, and overweight is defined as BMI > 25 Hazard ratios with 95% confidence intervals were adjusted for age, sex, income, smoking status, alcohol consumption status, regular exercise, hypertension, dyslipidemia, chronic kidney disease, cardiovascular disease, fasting glucose, duration of diabetes mellitus, use of insulin, and use of multiple (≥ 3) oral hypoglycemic agent SCA: sudden cardiac arrest; BMI: body mass index

Risk of SCA was further analyzed by subdividing body weight status into three groups: underweight, normal weight, and overweight. The risk of SCA was highest in sustained underweight (adjusted-HR = 2.433; 95% CI = 2.107–2.810), followed by those who had significant decrease of BMI over time: normal weight to underweight (adjusted-HR = 2.263; 95% CI = 1.999–2.563), and overweight to underweight (adjusted-HR = 2.253; 95% CI = 1.126–4.508; Fig. 3 and Additional Table 4). Risk of SCA was further compared according to the change of BMI at 4-year follow-up health examination. Becoming underweight in previously overweight patients was associated with significantly higher risk of SCA as compared with who remained overweight (*p* = 0.005) or became normal weight (*p* = 0.015, Fig. 3). In patients with normal weight, the risk of SCA was significantly higher in those who became underweight than those who remained normal weight (*p* < 0.001) or who became obese (*p* < 0.001).

**Fig. 4:**
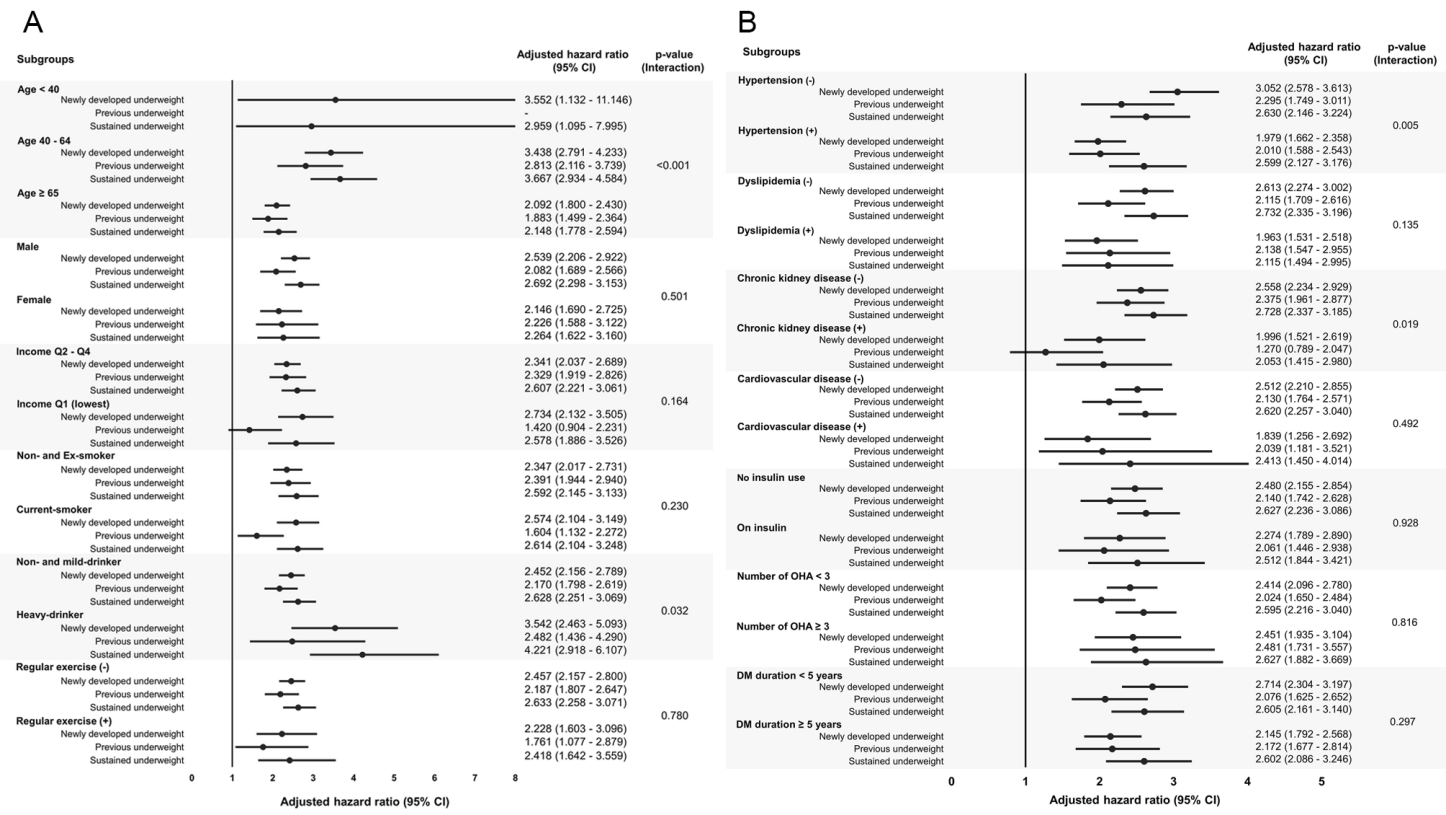
Subgroup analysis Sustained non-underweight was set as reference group, and was compared with newly developed underweight group, previous underweight group, and sustained underweight group. Hazard ratios with 95% confidence intervals were adjusted for age, sex, income, smoking status, alcohol consumption status, regular exercise, hypertension, dyslipidemia, chronic kidney disease, cardiovascular disease, fasting glucose, duration of diabetes mellitus, use of insulin, and use of multiple (≥ 3) oral hypoglycemic agent Q: quartile; CI: confidence interval; OHA: oral hypoglycemic agent; DM: diabetes mellitus

In subgroup analysis, subgroups with younger age, heavy drinkers, no hypertension or chronic kidney disease, presence of underweight at any time period was more strongly associated with SCA risk (Fig. 4).

## Discussion

**Fig. 5:**
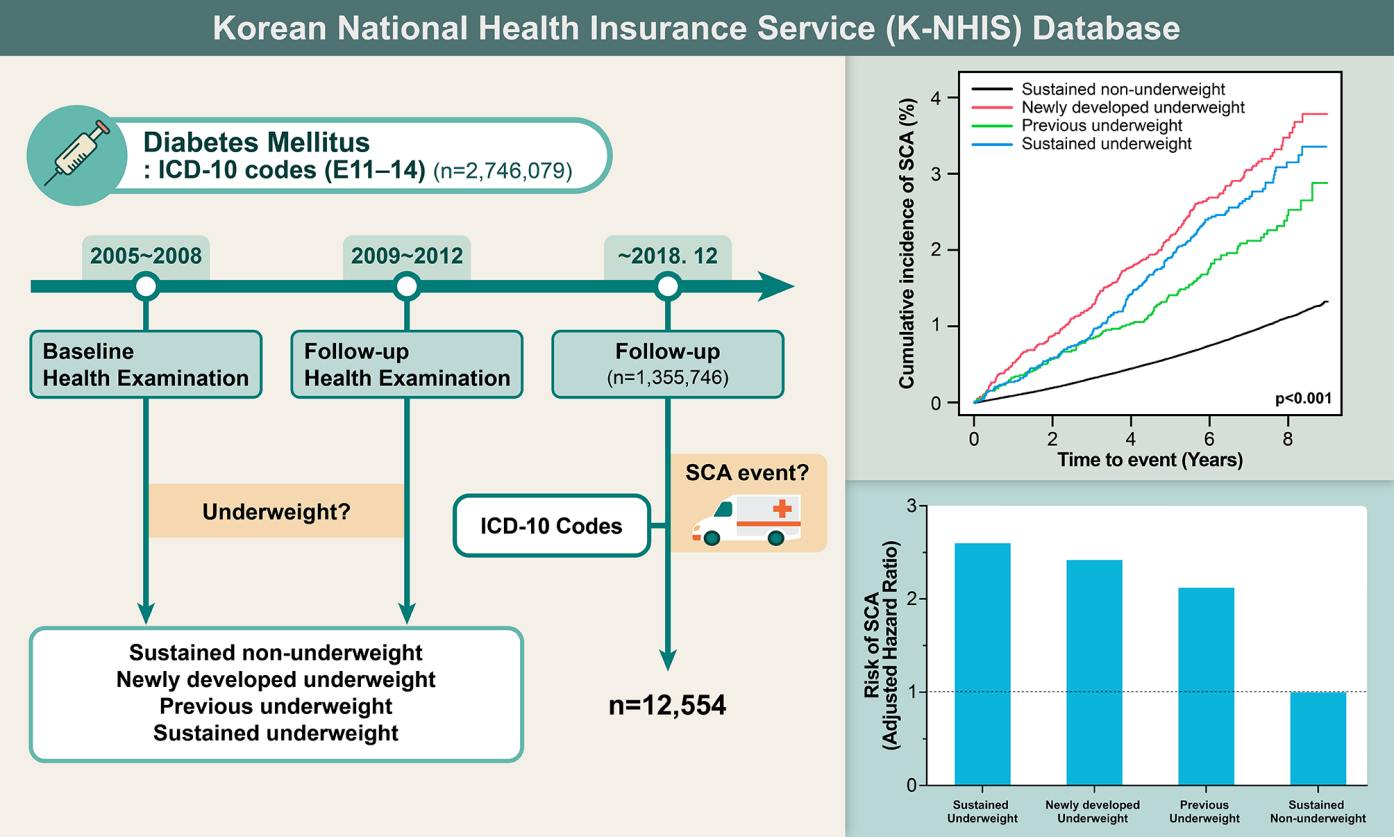
Impact of underweight and its temporal change on SCA risk ICD-10: International Classification of Disease, 10th revision; SCA: sudden cardiac arrest

This study investigated the association between temporal changes in BMI and the risk of SCA in patients with diabetes mellitus. Major findings from this study can be summarized as follows (Fig. 5). First, underweight at any time period is associated with significantly increased risk of SCA in patients with diabetes mellitus. Previous history of underweight is also associated with increased risk of SCA despite recovery to non-underweight. Second, recent development of underweight is associated with increased risk of SCA, regardless of previous body weight status. Third, sustained underweight both at baseline and follow-up is associated with highest risk of SCA. We comprehended large sample size of diabetes mellitus patients that underwent serial health examinations and analyzed sufficient cases of SCA (*n* = 12,554). Possible confounding factors related to body weight have been adjusted to clarify the association between SCA and underweight including its dynamic change.

### Underweight and dynamic change of body weight

We have previously identified underweight as an independent risk factor for SCA in diabetes mellitus [5]. However, body weight status can fluctuate during lifetime, and the influence of dynamic change in BMI on SCA risk was not clarified. Regarding the variability of body weight and the limitation of a single-time measurement, recent studies have focused on the longitudinal change of body weight [13, 14, 23, 24]. In patients with established coronary artery disease, fluctuations in body weight was strongly associated with cardiovascular events and mortality [13]. In addition, increase in body-weight variability was correlated with increased risk of adverse cardiovascular events. In prospective cohorts of general participants aged ≥ 45 years, risk of cardiovascular event and all-cause mortality was increased in both weight gain and weight loss, with higher risk found in weight loss group [24]. Similar finding was observed in patients with diabetes mellitus and higher cardiovascular risk, which resulted increased risk of heart failure or cardiovascular death in extreme weight gain or loss [14]. 

Our findings are in line with aforementioned previous studies. We found that patients who were in underweight at either baseline or follow-up health examination experienced significantly higher risk of SCA. Association between newly developed underweight over time (overweight to underweight, normal weight to underweight) and significantly increased risk of SCA suggests that decrease of body weight might causally influence the risk of SCA. On the other hand, substantially increased risk of SCA in diabetes mellitus patients who recovered to non-underweight from baseline underweight status suggests that adverse impact of underweight on SCA may not be fully reversible.

### Mechanisms

Several mechanisms support the association between SCA and underweight. First, patients with underweight exhibit decreased physiologic capacity and fat reserve, that may be more vulnerable to increase of metabolic demand such as critically ill condition. Malnutritional status also leads to decreased metabolic demands for oxygen delivery - such as decrease of plasma catecholamine or erythrocyte mass, and impairment of thyroxine deiodination - which may be more susceptible to acute illness that leads to SCA [25]. Second, underweight can be a result of a chronic progression of diabetes mellitus, that lead to systemic hypermetabolic condition. Newly developed underweight group had higher proportion of insulin use, multiple oral hypoglycemic agent administration, and prolonged diabetes mellitus. Uncontrolled diabetes mellitus can lead to various microvascular and macrovascular complications that involves major organs. In prolonged diabetes mellitus, coronary atherosclerosis is more accelerated, and renal function declined progressively. These patients with severe form of diabetes mellitus and underweight confront higher risk of coronary artery disease and renal dysfunction, which can increase the risk of SCA. Lastly, decrease of bodyweight may be caused by external condition such as malignancy, heart failure, respiratory disease, or systemic inflammatory disease. For instance, heart failure - with decreased cardiac output and increased venous pressure on gastrointestinal tract - may cause malnutrition, known as cardiac cachexia. Similarly, progressed systemic inflammatory disease that wastes muscle mass and decrease body weight may involve cardiopulmonary dysfunction. Thus, external conditions that influence body weight change may predispose to SCA.

### Clinical implication

In order to stratify the risk and prevent SCA, our findings can be adopted in clinical practice regarding patients with diabetes mellitus. First, underweight at any time period can increase the risk of SCA. Consequently, previously underweight patients who recovered to normal body weight should not be underestimated of SCA risk. Clinical factors and comorbidities that may be relevant to underweight should be assessed and modified. Second, decrease in bodyweight, especially to underweight, is associated with increased of SCA. Therefore, adequate nutritional support to avoid further deterioration of weight loss can be helpful to prevent SCA. Moreover, clinicians should monitor change in body weight status serially, and decrease in body weight should be paid with attention.

### Limitations

There are several limitations in this study. First, only patients that received sequential health examination in 4 years were included in the analysis, which may not be representative of all patients diagnosed as diabetes mellitus. However, the clinical differences between patients who were included and excluded (due to lack of sequential health examination) were not substantial. Moreover, we have excluded patients that lack sequential health examination data to minimize potential biases that could influence our findings. Second, possible confounding factors that might influence SCA might not be completely covered in our analysis. Although medications for diabetes mellitus and its duration were adjusted for analysis, concurrent degree of glucose intolerance or HbA1c was not comprehended. In addition, systemic illnesses that influence body weight status and SCA, such as respiratory disease and malignancy were not assessed. However, previous study of underweight that adjusted large volume of confounders resulted consistent effect of underweight on outcome, regardless of comorbidities, frailty measures, and markers of nutritional status [6]. Although more extensive adjustments of confounders were not performed in our study, increased SCA risk in underweight was robust. Third, primary outcome was defined as ICD-10 code-based SCA and consequences of SCA events were not assessed. Several studies that focused on clinical course of SCA have reported similar effect of lower BMI on mortality and neurologic outcomes [26–28]. Further study that focus on outcome after SCA in diabetes mellitus is needed.

## Conclusion

Sustained underweight as well as dynamic decrease in body weight are both significantly associated with increased risk of SCA, and recovery from underweight do not reduce the risk of SCA. In patients with diabetes mellitus, underweight at any time period can be associated with the SCA risk, which indicates appropriate recognition, modification of relevant conditions, as well as serial monitoring of body weight status.

### Electronic supplementary material

Below is the link to the electronic supplementary material.

Additional Material: Description of data: Additional Tables 1?4

## Data Availability

The data underlying this article are available in the article. The raw data underlying this article cannot be shared publicly due to privacy reasons and legal regulations of Republic of Korea. The raw data is stored and analyzed only in the designated server managed by the K-NHIS.

## Abbreviations

BMI: body mass index
CI: confidence interval
HR: hazard ratio
ICD-10: International Classification of Disease, 10th revision
K-NHIS: Korean National Health Insurance Service
SCA: sudden cardiac arrest
