# Supplementary material for: Association of temporal change in body mass index with sudden cardiac arrest in diabetes mellitus
Source: Cardiovasc Diabetol. 2024 Jan 28;23:46. doi: 10.1186/s12933-024-02130-4 (PMC10823669; doi:10.1186/s12933-024-02130-4)
Supplement: Supplementary file 1 — Additional Material: Description of data: Additional Tables 1?4 [file 12933_2024_2130_MOESM1_ESM.docx]

**ADDITIONAL MATERIAL**

**Association of Temporal change in Body Mass Index with Sudden Cardiac Arrest in Diabetes Mellitus**

**Additional Table 1. Definitions of variables**

**Additional Table 2. Baseline characteristics of study population and excluded population.**

**Additional Table 3. Comparison of baseline characteristics according to SCA**

**Additional Table 4. Impact of temporal change of BMI on SCA (Subdivided)**

**Additional Table 1. Definitions of variables**

| **Variables** | **Definition** |
| --- | --- |
| **Smoking** | |
| Non-smoker | <100 cigarettes in the lifetime |
| Ex-smoker | ≥100 cigarettes in the lifetime, but did not smoke within 1 month of health check-up in 2009 |
| Current smoker | ≥100 cigarettes in the lifetime, and continued smoking within 1 month of health check-up in 2009 |
| **Alcohol consumption** |  |
| Non-drinker | Those who consumed 0 g of alcohol per week |
| Mild-drinker | Those who consumed < 210 g of alcohol per week |
| Heavy-drinker | Those who consumed ≥ 210 g of alcohol per week |
| **Hypertension** | SBP ≥ 140 mmHg or DBP ≥ 90 mmHg or diagnostic codes for hypertension |
| **Regular exercise** | Those who had one or more sessions in a week with high (such as running, climbing, intense bicycle activities) or moderate physical activity (such as walking fast, tennis, or moderate bicycle activities) |
| **Cardiovascular disease** | Previous diagnosis of myocardial infarction or stroke |
| **Chronic kidney disease** | Estimated glomerular filtration rate < 60ml/min/1.73 m^2^ by the Modification of Diet in Renal Disease equation |

DBP, Diastolic blood pressure, mmHg; SBP, Systolic blood pressure, mmHg.

**Additional Table 2. Baseline characteristics of study population and excluded population.**

|  | **Included**  **(n=1,355,746)** | **Excluded**  **(n=1,306,520)** | **p-value** |
| --- | --- | --- | --- |
| **Age groups** |  |  | <.001 |
| <40 | 89,630 (6.6%) | 109,946 (8.4%) |  |
| 40-64 | 820,631 (60.5%) | 821,164 (62.9%) |  |
| ≥65 | 445,485 (32.9%) | 375,410 (28.7%) |  |
| **Sex** |  |  | <.001 |
| Male | 840,676 (62.0%) | 759,000 (58.1%) |  |
| Female | 515,070 (38.0%) | 547,520 (41.9%) |  |
| **Income, Lowest Quartile** | 239,602 (17.7%) | 313,970 (24.5%) | <.001 |
| **Smoking** |  |  | <.001 |
| Non-smoker | 756,400 (55.8%) | 720,249 (55.3%) |  |
| Ex-smoker | 277,596 (20.5%) | 216,361 (16.6%) |  |
| Current smoker | 321,750 (23.7%) | 366,825 (28.1%) |  |
| **Drinking** |  |  | <.001 |
| Non-drinker | 772,849 (57.0%) | 751,318 (57.9%) |  |
| Mild-drinker | 458,663 (33.8%) | 406,398 (31.3%) |  |
| Heavy-drinker | 124,234 (9.2%) | 139,744 (10.8%) |  |
| **Regular exercise** | 303,931 (22.4%) | 242,042 (18.6%) | <.001 |
| **Hypertension** | 782,576 (57.7%) | 737,722 (56.5%) | <.001 |
| **Dyslipidemia** | 579,614 (42.8%) | 545,964 (41.8%) | <.001 |
| **Chronic kidney disease** | 82,788 (6.1%) | 82,324 (6.3%) | <.001 |
| **Cardiovascular disease** | 155,635 (11.5%) | 155,643 (11.9%) | <.001 |
| **Diabetes mellitus Duration, ≥5 years** | 462,141 (34.1%) | 392,715 (30.1%) | <.001 |
| **Use of insulin** | 115,422 (8.5%) | 126,956 (9.7%) | <.001 |
| **Use of oral hypoglycemic agents, ≥ 3** | 204,255 (15.1%) | 187,484 (14.4%) | <.001 |
| **Age, years** | 58.5 ± 11.8 | 56.7 ± 13.0 | <.001 |
| **Body mass index, kg/m^2^** | 25.0 ± 3.3 | 25.1 ± 4.0 | <.001 |
| **Waist Circumference, cm** | 85.4 ± 8.4 | 85.4 ± 9.0 | <.001 |
| **Systolic Blood Pressure, mmHg** | 128.7 ± 15.3 | 129.3 ± 16.4 | <.001 |
| **Diastolic Blood Pressure, mmHg** | 78.8 ± 10.0 | 79.2 ± 10.6 | <.001 |
| **Fasting glucose, mg/dL** | 141.6 ± 43.7 | 148.0 ± 50.6 | <.001 |
| **Total cholesterol, mg/dL** | 194.6 ± 41.8 | 197.5 ± 44.4 | <.001 |
| **HDL -cholesterol, mg/dL** | 51.7 ± 22.6 | 52.1 ± 24.9 | <.001 |
| **LDL -cholesterol, mg/dL** | 110.2 ± 40.3 | 111.2 ± 41.4 | <.001 |
| *** Triglyceride, mg/dL** | 143.9 (143.8-144.3) | 149.8 (149.7-150.0) | <.001 |
| **Incidence rate of SCA** | 1.648 | 1.377 |  |

Patients who were included in the analysis (Included, n=1,355,746) were compared to those who did not undergo baseline health examination at 2005-2008 (Excluded, n=1,306,520).

HDL, high-density lipoprotein; LDL, low-density lipoprotein; SCA, sudden cardiac arrest.

**Additional Table 3. Comparison of baseline characteristics according to SCA**

|  | **Sudden Cardiac Arrest** | | **p-value** |
| --- | --- | --- | --- |
|  | **No (n=1,343,192)** | **Yes (n=12,554)** |  |
| **Age groups** |  |  | <.001 |
| <40 | 89,481 (6.7%) | 149 (1.2%) |  |
| 40-64 | 815,910 (60.7%) | 4,721 (37.6%) |  |
| ≥65 | 437,801 (32.6%) | 7,684 (61.2%) |  |
| **Sex** |  |  | <.001 |
| Male | 831,429 (61.9%) | 9,247 (73.7%) |  |
| Female | 511,763 (38.1%) | 3,307 (26.3%) |  |
| **Income, Lowest Quartile** | 237,085 (17.7%) | 2,517 (20.1%) | <.001 |
| **Smoking** |  |  | <.001 |
| Non-smoker | 750,205 (55.9%) | 6,195 (49.4%) |  |
| Ex-smoker | 274,645 (20.5%) | 2,951 (23.5%) |  |
| Current smoker | 318,342 (23.7%) | 3,408 (27.2%) |  |
| **Drinking** |  |  | <.001 |
| Non-drinker | 764,957 (57.0%) | 7,892 (62.9%) |  |
| Mild-drinker | 455,134 (33.9%) | 3,529 (28.1%) |  |
| Heavy-drinker | 123,101 (9.2%) | 1,133 (9.0%) |  |
| **Regular exercise** | 301,338 (22.4%) | 2,593 (20.7%) | <.001 |
| **Hypertension** | 773,548 (57.6%) | 9,028 (71.9%) | <.001 |
| **Dyslipidemia** | 574,278 (42.8%) | 5,336 (42.5%) | 0.573 |
| **Chronic kidney disease** | 152,581 (11.4%) | 3,054 (24.3%) | <.001 |
| **Cardiovascular disease** | 81,388 (6.1%) | 1,400 (11.2%) | <.001 |
| **Diabetes mellitus Duration, ≥5 years** | 455,843 (33.9%) | 6,298 (50.2%) | <.001 |
| **Use of insulin** | 113,012 (8.4%) | 2,410 (19.2%) | <.001 |
| **Use of oral hypoglycemic agents, ≥ 3** | 201,549 (15.0%) | 2,706 (21.6%) | <.001 |
| **Age, years** | 58.5 ± 11.8 | 66.1 ± 10.5 | <.001 |
| **Body mass index, kg/m^2^** | 25.0 ± 3.3 | 24.3 ± 3.6 | <.001 |
| **Waist Circumference, cm** | 85.4 ± 8.4 | 86.0 ± 8.8 | <.001 |
| **Systolic Blood Pressure, mmHg** | 128.7 ± 15.3 | 130.8 ± 17.2 | <.001 |
| **Diastolic Blood Pressure, mmHg** | 78.8 ± 10.0 | 78.3 ± 10.9 | <.001 |
| **Fasting glucose, mg/dL** | 141.6 ± 43.6 | 144.5 ± 54.3 | <.001 |
| **Total cholesterol, mg/dL** | 194.7 ± 41.8 | 189.3 ± 44.3 | <.001 |
| **HDL -cholesterol, mg/dL** | 51.7 ± 22.6 | 49.9 ± 22.1 | <.001 |
| **LDL -cholesterol, mg/dL** | 110.2 ± 40.3 | 107.3 ± 42.6 | <.001 |
| *** Triglyceride, mg/dL** | 143.9 (143.8-144.1) | 140.8 (139.5-142.2) | <.001 |

* expressed as median (interquartile range)

SCA, sudden cardiac arrest; HDL, high-density lipoprotein; LDL, low-density lipoprotein.

**Additional Table 4. Impact of temporal change of BMI on SCA (Subdivided)**

| **BMI, kg/m^2^** | | **N** | **Event** | **Duration** | **IR, per 1000** | **Adjusted HR (95% Confidence Interval)** | | | |
| --- | --- | --- | --- | --- | --- | --- | --- | --- | --- |
| **Baseline** | **4 years later** |  |  |  |  | **Model 1** | **Model 2** | **Model 3** | **Model 4** |
| <18.5 | <18.5 | 8,162 | 193 | 49,487 | 3.90002 | 2.546 (2.206 - 2.939) | 2.409 (2.087 - 2.781) | 2.438 (2.111 - 2.816) | 2.433 (2.107 - 2.810) |
|  | <25 | 6,213 | 121 | 39,308 | 3.07822 | 1.996 (1.668 - 2.390) | 2.066 (1.726 - 2.474) | 2.046 (1.709 - 2.450) | 2.009 (1.678 - 2.406) |
|  | ≥25 | 202 | 2 | 1,331 | 1.50261 | 0.966 (0.241 - 3.862) | 0.961 (0.240 - 3.845) | 0.882 (0.220 - 3.527) | 0.934 (0.233 - 3.735) |
| <25 | <18.5 | 9,985 | 262 | 58,997 | 4.44087 | 2.916 (2.576 - 3.300) | 2.417 (2.135 - 2.736) | 2.397 (2.117 - 2.714) | 2.263 (1.999 - 2.563) |
|  | <25 | 562,172 | 5,855 | 3,763,621 | 1.55568 | 1 (Reference) | 1 (Reference) | 1 (Reference) | 1 (Reference) |
|  | ≥25 | 86,527 | 761 | 585,534 | 1.29967 | 0.835 (0.774 - 0.900) | 0.919 (0.852 - 0.991) | 0.885 (0.821 - 0.955) | 0.900 (0.834 - 0.971) |
| ≥25 | <18.5 | 285 | 8 | 1,624 | 4.92397 | 3.231 (1.619 - 6.450) | 2.482 (1.240 - 4.966) | 2.355 (1.177 - 4.712) | 2.253 (1.126 - 4.508) |
|  | <25 | 116,902 | 1,163 | 780,967 | 1.48918 | 0.958 (0.899 - 1.020) | 0.981 (0.921 - 1.044) | 0.950 (0.892 - 1.011) | 0.948 (0.890 - 1.010) |
|  | ≥25 | 565,298 | 4,189 | 3,834,220 | 1.09253 | 0.702 (0.675 - 0.730) | 0.838 (0.806 - 0.872) | 0.801 (0.769 - 0.834) | 0.831 (0.798 - 0.866) |

BMI, body mass index; SCA, sudden cardiac arrest; IR, incidence rate; HR, hazard ratio.

Model 1: non-adjusted

Model 2: adjusted for age and sex.

Model 3: adjusted for age, sex, income, smoking status, alcohol consumption status, regular exercise, hypertension, dyslipidemia, chronic kidney disease, cardiovascular disease.

Model 4: adjusted for age, sex, income, smoking status, alcohol consumption status, regular exercise, hypertension, dyslipidemia, chronic kidney disease, cardiovascular disease, fasting glucose, duration of diabetes mellitus, use of insulin, use of oral hypoglycemic agent.
